# The Early Worm Catches the Bird? Productivity and Patterns of *Trichobilharzia szidati* Cercarial Emission from *Lymnaea stagnalis*

**DOI:** 10.1371/journal.pone.0149678

**Published:** 2016-02-19

**Authors:** Miroslava Soldánová, Christian Selbach, Bernd Sures

**Affiliations:** 1 Institute of Parasitology, Biology Centre of the Czech Academy of Sciences, České Budějovice, Czech Republic; 2 Department of Aquatic Ecology and Centre for Water and Environmental Research (ZWU), University of Duisburg-Essen, Essen, Germany; 3 Department of Zoology, University of Johannesburg, Johannesburg, South Africa; George Washington University School of Medicine and Health Sciences, UNITED STATES

## Abstract

Digenean trematodes are common and abundant in aquatic habitats and their free-living larvae, the cercariae, have recently been recognized as important components of ecosystems in terms of comprising a significant proportion of biomass and in having a potentially strong influence on food web dynamics. One strategy to enhance their transmission success is to produce high numbers of cercariae which are available during the activity peak of the next host. In laboratory experiments with 13 *Lymnaea stagnalis* snails infected with *Trichobilharzia szidati* the average daily emergence rate per snail was determined as 2,621 cercariae, with a maximum of 29,560. During a snail’s lifetime this summed up to a mass equivalent of or even exceeding the snail’s own body mass. Extrapolated for the eutrophic pond where the snails were collected, annual *T*. *szidati* biomass may reach 4.65 tons, a value equivalent to a large Asian elephant. Emission peaks were observed after the onset of illumination, indicating emission synchronizing with the high morning activities of the definitive hosts, ducks. However, high cercarial emission is possible throughout the day under favorable lightning conditions. Therefore, although bird schistosomes, such as *T*. *szidati* constitute only a fraction of the diverse trematode communities in the studied aquatic ecosystem, their cercariae can still pose a considerable risk for humans of getting cercarial dermatitis (swimmer's itch) due to the high number of cercariae emitted from infected snails.

## Introduction

Except for their role as pathogens, parasites have long been considered negligible components of ecosystems. Recent studies elucidating the patterns of parasite biomass, species abundance and interactions in food webs have revealed that parasites actually represent important ecological players in the dynamics of natural systems [1–5]. Especially free-swimming larvae of trematodes, the cercariae, which emerge from the molluscan first intermediate hosts as a result of asexual reproduction, represent essential components of ecosystems subsuming a substantial fraction of biomass in marine [3,6] and freshwater ecosystems [5,7] and may exert strong influence on the structure, dynamics and function of food webs [1,2,8,9].

Digenean trematodes have complex life cycles which usually involve three hosts mutually connected through various trophic relations. Trematodes require molluscs as their first intermediate hosts, which become infected by penetration of free-swimming miracidia hatched from eggs. Within the snail host, the trematodes develop into sporocysts and/or rediae and asexually produce numerous dispersal larvae, the cercariae, which are emitted into the aquatic environment. After penetrating a suitable second intermediate host, which belongs to a broad spectrum of invertebrates and vertebrates, the cercariae encyst as metacercariae and are usually consumed by their definitive host, mostly a vertebrate, where they develop into adults, mate and produce eggs. Accordingly, the cercarial stage is crucial in the life cycle of trematodes as it plays an inevitable role in the transmission to the next target hosts. After emerging from an infected snail host, the principal task of cercariae is to disperse in the environments, locate and infect as many hosts as possible. However, only a small fraction of the emitted cercariae successfully reaches its target because cercariae are short-lived and directly exposed to and affected by external biotic and abiotic environmental factors often leading to a failure in infecting the next host [10,11]. Accordingly, many trematode species produce high numbers of cercariae, whose shedding peak is assumed to be synchronized with the behavior of the next host [12] and is often triggered by factors such as the photoperiod and light exposure or water temperature [13,14].

**Fig 1:**
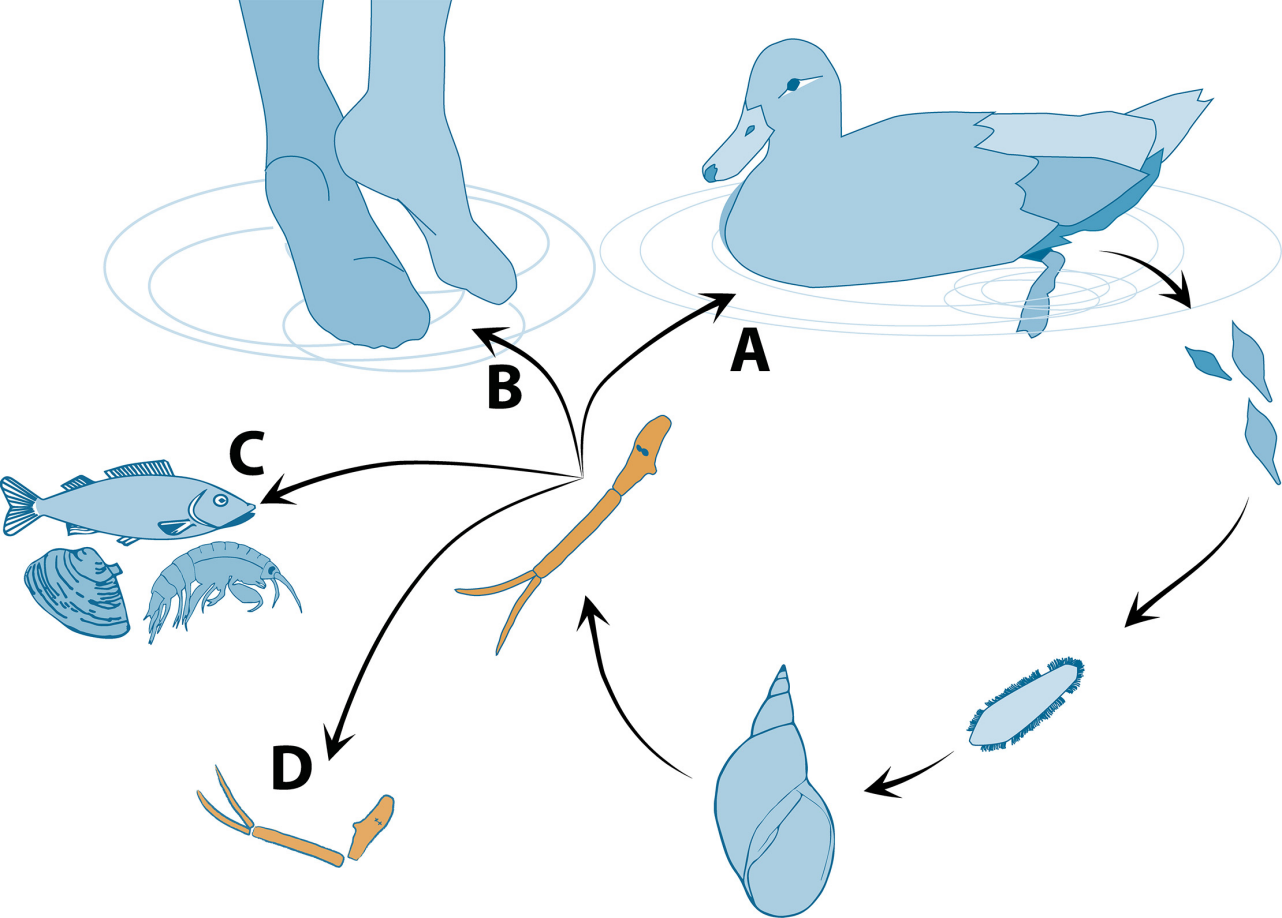
Life-cycle of *Trichobilharzia szidati* showing primary and secondary roles of its free-living stages, the cercariae. **(A)** Infection of suitable definitive bird host, **(B)** infection of humans as accidental host, causing swimmer’s itch, **(C)** source of food to a wide range of organisms in local communities as prey items or **(D)** becoming a part of detrital food webs.

However, in some trematode species these free-living cercariae are of medical importance as causative agents of important diseases. In Europe and North America, the cercariae of bird schistosomes (family Schistosomatidae) of the genus *Trichobilharzia* are the most common and important agents of swimmer’s itch, an inflammatory skin reaction [15,16]. Bird schistosomes of the genus *Trichobilharzia* possess a two-host life cycle utilizing freshwater pulmonate snails and waterfowl as first intermediate and definitive hosts, respectively. Once released from the snail, the cercariae actively seek out a suitable definitive host, which they infect directly (Fig 1A). However, since cercariae rely on general host-finding stimuli (e.g. water turbulences or chemical cues), they may accidentally infect humans where they can cause swimmer’s itch (Fig 1B) [15,17]. It can be assumed, that only few cercariae will find a suitable final bird host or infect an accidental human host whereas the majority of individuals are likely to serve as food for predators or contribute to the ecosystem’s detritus after their limited life span (Fig 1C and 1D), thereby playing a significant role in ecosystem energetics.

Although complex information about various aspects of the biology of *Trichobilharzia* spp. has been gathered in past decades [17–21] available data on the patterns of cercarial emission are still rather fragmentary and virtually no reliable data exist on the total number of cercariae entering the ecosystem. Early observations and estimations of cercarial productivity of *Trichobilharzia* are rather broad, ranging from several thousand cercariae during a snail’s lifetime [22] to several thousand larvae during a few days or weeks [23,24]. Likewise, temporal emergence patterns have been studied for a variety of trematode species [12], but there are only few studies investigating the temporal patterns for *Trichobilharzia* sp. cercariae in detail [23] that allow to assess these patterns on an ecosystem level.

Therefore, the main aim of the present study was twofold. First, to examine cercarial emergence of the model organism of bird schistosomes, *Trichobilharzia szidati*, from its specific host *Lymnaea stagnalis* under laboratory conditions in order to determine (i) daily output rates per individual snail; (ii) peaks in cercarial emission under controlled and natural light conditions; and (iii) output variation among conditions and experiments. Accordingly, we investigated cercarial emergence in a series of laboratory experiments under different conditions using 13 naturally infected *L*. *stagnalis* with *T*. *szidati* over a 72-hour period during each experiment. The second goal was to assess the direct contribution of the parasite larvae to ecosystem energetics using estimates for cercarial abundance and biomass of *T*. *szidati*. We quantified cercarial biomass using data on mean daily output rates and metrical data of living cercariae, and finally we estimated the total biomass of cercariae for the life span of an individual snail as well as for the snail population within a typical freshwater ecosystem in Europe using data from the literature on snail abundance and *Trichobilharzia* spp. prevalence. In light of recent investigations on trematode biomass, we were particularly interested in the overall significance of cercarial numbers and biomass estimates of a single life stage of a single, medically important, trematode species for the aquatic ecosystem, regarding food web dynamics, energy transfer, parasite transmission and disease risk in humans. As there are several barriers on the way preventing successful completion of the parasite’s life cycle, quantifying the total parasite output during the host’s life span and its biomass in the environment may have important implications for our understanding of the role of cercariae of *T*. *szidati* at multiple levels in the ecosystem, well beyond their usually discussed role as disease agents of swimmer’s itch.

## Materials and Methods

### Experimental design

In our experimental study we selected *Trichobilharzia szidati* Neuhaus, 1952 as a model organism because knowledge on exact total or average numbers of cercariae released from a snail host is rather ambiguous and quantitative estimates on its cercariae productivity is entirely lacking, although it is probably the most studied model bird schistosome [18,25]. Cercariae of *T*. *szidati* were obtained from naturally infected *Lymnaea stagnalis* (L.) which were sampled from a fishpond in the Czech Republic in June 2012 (Vlkovský pond: 49°08'56"N, 14°43'51"E). A total of 168 snails was collected along the pond shore from aquatic vegetation, transported to the laboratory and screened for patent infections by placing them individually into beakers with a small amount of filtered lake water under the light source for 24 hours. To avoid the possible influence of different snail host sizes on the cercarial emergence when larger snails produce higher daily outputs [11,13,26], sampling effort was focused on snails of similar size cohorts between 40–50 mm in shell height only. Moreover, in this size/age *L*. *stagnalis* can be found with higher percentage of infection [27] assuring a higher probability of encountering the desired trematode species. In total, 13 snails with *T*. *szidati* infections were found (7.74% of 168 snails examined). Because several stress factors such transportation of snails and their subsequent handling under different temperatures and illumination scenarios in the laboratory might negatively affect both host and parasites leading to distorted rhythms of cercarial emergence [14,23,28,29], all snails infected with *T*. *szidati* were maintained in one aerated aquarium at room temperature ranging from 19°C to 21°C and under natural photoperiod for 14 days for acclimatization. Snails were stopped feeding on lettuce five days prior to emission experiments and were measured before each experiment. As ambient temperature is considered the most important factor influencing daily cycles of cercarial emergence [13] all experiments were designed under stable thermal conditions and both air and water temperature were monitored throughout the entire course of the study.

Cercarial emergence of *T*. *szidati* from 13 naturally infected *L*. *stagnalis* was investigated under different conditions in a series of two types of laboratory experiments, each performed over three consecutive days in July and September (i.e. for 72 hours). A “daily output experiment” was designed to obtain data on the total and mean number of cercariae released per snail and day. Cercarial counts were carried out every four hours from 8:00 to 20:00 over a period of 72 hours. Because examination of cercariae for the state of degradation did not reveal any decomposition during this period, cercariae produced during the night intervals were counted once in the morning after 12-hours (from 20:00 to 8:00). Additionally, “peak output experiments” were performed aiming at assessing the chronobiological variation in a cercarial release during 72 hours, and to determine the day period with the highest emission rates. Cercarial counts were performed every two hours over a period of 72 hours. Each type of experiment was conducted under two different laboratory conditions, a natural photoperiod regime (sunrise at 5:30 and sunset at 21:50 in July; i.e. 16:20 h of light and 7:40 h of darkness) in the laboratory at room temperature (range from 19.6°C to 20.4°C; mean 20.2°C), and 12:12 light-dark cycle under standard conditions in a climate chamber with the temperature set to 20°C. In the climate chamber illumination was provided by an overhead halogen lamp; in the laboratory, snails were exposed to the natural photoperiod regime by placing them next to the window. In total, we investigated variation in cercarial output in four consecutive experiments in July: one “daily output experiment” at room temperature (further DJ-L) and one in a climate chamber (DJ-C), further one “peak output experiment” at room temperature in the laboratory (further PJ-L) and one in a climate chamber (PJ-C) (Table 1). Altogether, the four experiments in July lasted 15 days with an interval of one free day in between experiments. In September, only one “peak output experiment” in the climate chamber (PS-C) was performed (Table 1). Light dark circles in the climate chamber were set from 6:00 to 18:00 light and 18:00 to 6:00 dark in July and 8:00 to 20:00 light and 20:00 to 8:00 dark in September. No specific permissions were required for these locations/activities and our field study does not involve endangered or protected species.

**Table 1:** Numbers of cercariae of *Trichobilharzia szidati* released from naturally infected *Lymnaea stagnalis* per snail.

	**July**												**September**		
Snailcode	Daily output experiment in laboratory (DJ-L; n = 13)			Peak output experiment in laboratory (PJ-L; n = 11)			Peak output experiment in climate chamber (PJ-C; n = 11)			Daily output experiment in climate chamber (DJ-C; n = 10)			Peak output experiment in climate chamber (PS-C; n = 9)		
	Range	Mean±SD	Total	Range	Mean±SD	Total	Range	Mean±SD	Total	Range	Mean±SD	Total	Range	Mean±SD	Total
L1	510–2,740	1,483±1,142	4,450	1,290–2,520	1,700±710	5,100	1,650–3,230	2,190±901	6,570	400–2,320	1,603±1,009	4,810	9,550–24,070	15,213±7,769	45,640
L2	1,310–1,500	1,390±98	4,170	1,890–2,510	2,160±318	6,480	1,870–3,390	2,393±864	7,180	560–2,080	1,107±845	3,320	7,530–21,430^b^	13,003±7,406	39,010
L3	360–620	460±140	1,380	810–1,760	1,190±503	3,570	430–1,100	667±376	2,000	390–1,000	703±305	2,110	2,520–7,920^b^	4,947±2,741	14,840
L4	330–1,020	597±371	1,790	740–1,680	1,300±495	3,900	520–730	590±121	1,770	40–260	173±117	520	5,970–19,950	12,783±6,997	38,350
L5	980–1,480	1,150±286	3,450	810–990	903±90	2,710	910–1,600	1,147±393	3,440	260–620	453±181	1,360	2,620–14,750^b^	10,470±6,808	31,410
L6	600–1,480	1,143±475	3,430	860–2,590	1,647±876	4,940	1,580–2,940	2,083±746	6,250	700–1,880	1,093±681	3,280	6,510–29,560	17,947±11,526	53,840
L7	380–1,490	963±557	2,890	1,360–2,600	1,887±641	5,660	240–2,230	960±1,103	2,880	100–140	120±20	360	2,690–4,720^b^	3,707±1,015	11,120
L8	540–1,200	880±330	2,640	880–1,450	1,197±290	3,590	350–780	553±216	1,660	- ^a^	-	-	-^a^	-	-
L9	780–1,190	1,043±229	3,130	1,160–1,350	1,277±102	3,830	910–2,060	1,457±577	4,370	560–1,820	1,117±643	3,350	5,290–10,730	7,993±2,720	23,980
L10	550–1,620	1,203±573	3,610	-^a^	-	-	-^a^	-	-	-	-	-	-^a^	-	-
L11	300–690	490±195	1,470	610–1,590	1,007±516	3,020	390–2,130	1,057±939	3,170	330–1,640	1,070±671	3,210	2,840–8,730^b^	5,197±3,116	15,590
L12	940–3,000	1,740±1,104	5,220	- ^a^	-	-	-^a^	-	-	-	-	-	-^a^	-	-
L13	580–4,560	1,927±2,281	5,780	600–1,800	1,267±611	3,800	360–1,940	987±839	2,960	220–2,700	1,120±1,373	3,360	-^a^	-	-

Range, means (± standard deviation, SD) and total numbers of emerged cercariae are given per snail pooled across three days (i.e. 72 hours) of each experiment. In September only one experiment was performed. Number of snails used in a given experiment is indicated by “n” in parentheses.

^a^Snail died during the experiment.

^b^Snail with patent double infection.

### Determining cercarial numbers

The emission experiment of cercariae from individual snail replicates was carried out in plastic cups with 100 ml of lake water. Prior to use, the water was filtered in order to avoid contamination and placed in the relevant conditions corresponding to each type of experiment to balance potential differences in temperature. After each emission period, ten homogenized subsamples of one ml each were taken with a micropipette from each replicate while vigorously mixing the water containing swimming larvae and transferred into cell-well plates to count cercariae. Drops of vital stain (Natural Red) were added to make cercariae immobile and more visible, allowing precise counts under a dissection microscope. After each emission time unit interval (two or four and 12 hours depending on the type of experiment), snails were transferred to new clean plastic cups with fresh pond water. To avoid contamination of the sample with cercariae of other replicates, the spoon used was washed and dried thoroughly. Raw data (i.e. counts per snail per unit time) for each replicate were converted into daily output rates (i.e. number of cercariae emitted snail^-1^ day^-1^) as follows: the numbers of cercariae found in one ml after each emission interval were summed over 10 subsamples and means were calculated. Averages were multiplied by 100 (water volume) and pooled across all emission intervals from one day, resulting in an estimate of the total number of cercariae emitted from a single snail during a day.

### Estimation of cercarial biomass

In order to assess the direct contributions of *T*. *szidati* cercariae to the energy flow in ecosystems, its cercarial biomass was quantified using data on the mean daily output rates which we acquired from emission experiments, metrical data of live cercariae and data from literature on snail abundance and parasite prevalence. First, we obtained metrical data of 11 unflattened live cercariae by measuring length and width of cercarial body, tail stem and furcae from photographs taken with an Olympus UC30 digital camera fitted on an Olympus BX51 microscope. Measurements were taken with the program ImageJ 1.47v [30]. Based on formulas provided by Koehler et al. [31] we calculated the total cercarial volume (in mm^3^) as sum of volumes calculated for the cercarial body (equation for ellipsoid), tail stem (equation for cylinder) and furcae (equation for cone), and estimated mass (in mg) of an individual cercaria by multiplying the total cercarial volume by tissue density of 1.1 g/ml [3]. Thereafter, we estimated parasite productivity for the life span of an individual snail (2 years for *L*. *stagnalis*) [32], considering the mean cercarial emission per snail and day (pooled across all five experiments) and the hibernation period of *L*. *stagnalis* in moderate climate of Central Europe. Given that development of many trematode species in snails throughout the winter period is arrested and no cercarial emergence occurs due to the decreased metabolic activity of both host and parasite [33], we assume that the ongoing uninterrupted cercarial emission of *T*. *szidati* persists from April to October in two subsequent years (428 days). Several studies reported spontaneous loss of trematode infections in snail hosts (i.e. “self-cure”), predominantly in freshwater systems ([34]). Although these extinction events were recorded for several trematode species including *T*. *szidati*, in eutrophic pond systems extinction rates were substantially lower than colonisation rates (i.e. new infections in snails) and thus not considered high enough to significantly affect prevalence patterns in the snail populations [35]. Therefore, we assumed that an established infection of *T*. *szidati* persists during the life span of the snail host and thus cercariae are emitted continuously as long as the snail lives (except for winter months). The total mass of *T*. *szidati* emitted from one infected snail during its life span was calculated by multiplying cercarial mass by mean daily cercarial output per snail by 428 days. The cercarial mass estimated to be emitted from a single snail individual was afterwards compared with the tissue mass of 30 uninfected snails of similar size (mean of 45.1 mm) to those 13 snails infected with *T*. *szidati* used in the experiments (mean of 42.2 for snails entering experiments in July and 47.5 mm for snails in September), which we obtained by weighing snail body deprived of their shells. Furthermore, we were interested in the total annual biomass of *T*. *szidati* cercariae entering an ecosystem. Accordingly, we used data from the literature on density of *L*. *stagnalis* (10 snails/m^2^) [36] to estimate the total biomass of cercariae in the pond from which the snails were collected. We assessed the snail population size within the ecosystem by multiplying the snail density of 10 snails/m^2^ by surface area (m^2^) of the water body (47.09 ha for Vlkovský pond). Considering the usual prevalence of *T*. *szidati* in snail populations in Europe (5%) [15] we estimated the total number of infected snails within the water body and calculated the total biomass of cercariae released by these snails during one year only from April to October (214 days). Since infection levels of *T*. *szidati* in eutrophic ponds might exceed 20% and occasionally reach more than 40% [15] we calculated a biomass of cercariae in the pond estimating a prevalence of 41.5% [15,37].

### Data analyses

We used the non-parametric Spearman's correlation coefficient (r_s_) to statistically assess the effect of host size (shell height) on cercarial emergence. Correlation was tested for total numbers of cercariae pooled across three days (i.e. 72 hours) and by each day of all five experiments separately due to unequal numbers of replicates. Due to the one “peak emission experiment” conducted in September (PS-C), cercarial numbers were compared with only two experiments of equal treatment in July, i.e. PJ-L and PS-C; and PJ-C and PS-C. Furthermore, five snails in the September experiment showed patent double infections with other trematode species (four with *Diplostomum pseudospathaceum* and one with *Plagiorchis elegans*). Therefore, Student’s t-test was applied to detect significant difference in total numbers of *T*. *szidati* cercariae (pooled across three days) emitted from singly and doubly infected snails.

To assess output variation among experiments in different conditions we carried out a comparative statistical assessment on daily cercarial emergence using a general linear model (GLM) repeated measures ANOVA (RM-ANOVA) with cercarial numbers as dependent variable and “experiment” and “day” as within-subjects factors. Further, we analyzed whether there are significant differences in cercarial output between all three “peak output experiments” (PJ-L, PJ-C and PS-C) in relation to time unit interval of two hours and day of experiment (“hour” and “day” as within-subjects factors). Post hoc Tukey HSD tests were performed where appropriate. Data on cercarial counts were ln (x+1)-transformed in order to meet the assumption of normality. A probability value of p< 0.05 was considered to represent a significant difference in all comparisons. Statistical analyses were performed using Statistica 7.0 software package (StatSoft, Inc., Tulsa, OK, USA).

## Results

### Emergence and biomass of cercariae

**Fig 2:**
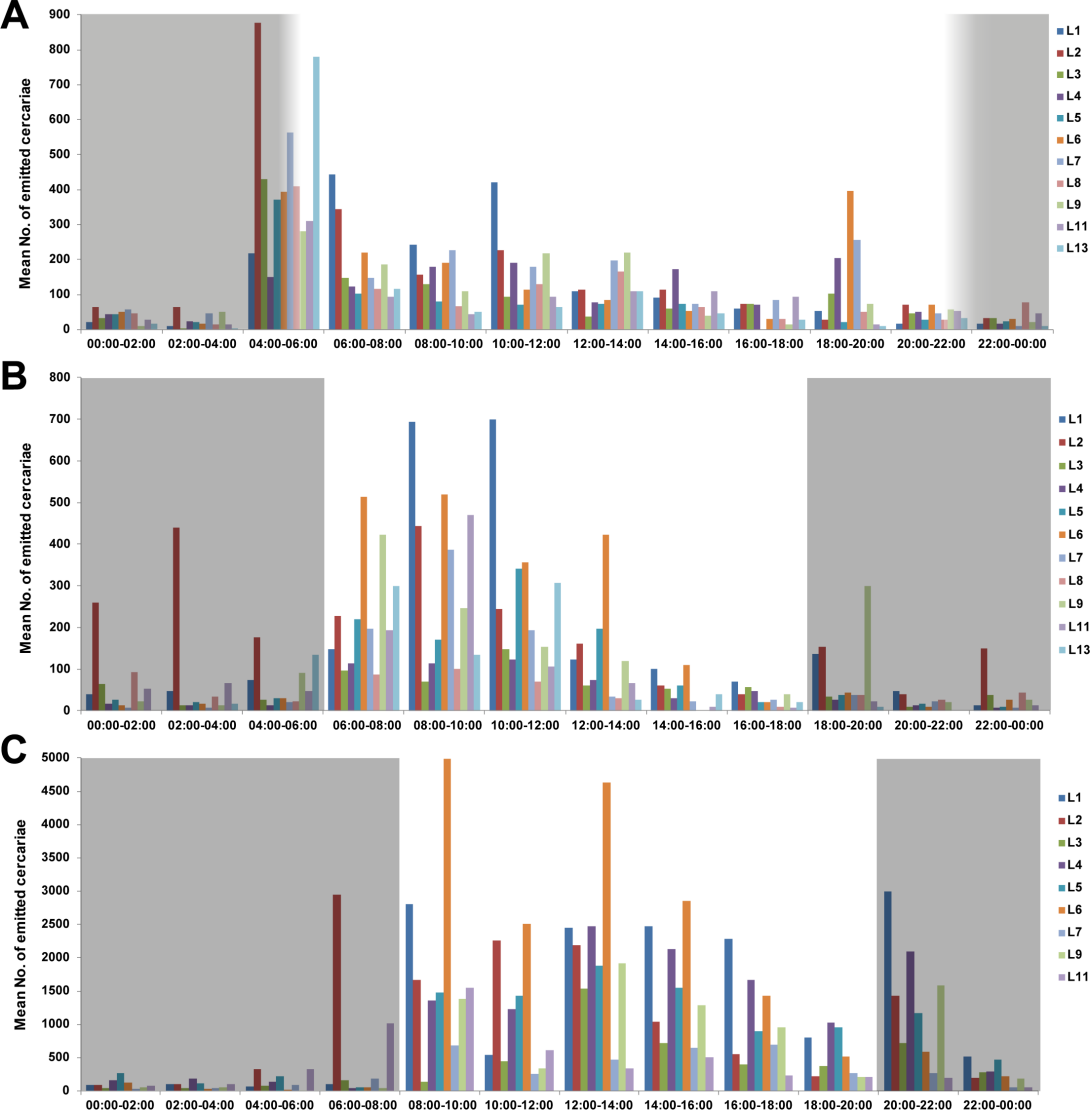
Daily output rates and emergence patterns of *Trichobilharzia szidati* cercariae from *Lymnaea stagnalis* individuals. Plots show circadian cercarial emission with high levels in the light part of the day and variability across individual snail replicates (L1–13). Data are pooled across three days of each experiment. Grey areas indicate dark periods of the day. **(A)** Mean number of emitted cercariae from 11 naturally infected snails used in a “peak emission experiment” (PJ-L) in July under laboratory conditions at room temperature (range 19.6°C-20.4°C) and natural photoperiod regime (light dark cycle of 16:8 h). **(B)** Mean number of emitted cercariae from 11 naturally infected snails used in a “peak emission experiment” (PJ-C) in July under standard thermal conditions in a climate chamber (20°C) and controlled light dark cycle of 12:12 h. **(C)** Mean number of emitted cercariae from 9 naturally infected snails used in a “peak emission experiment” (PS-C) in September under standard thermal conditions in a climate chamber (20°C) and controlled light dark cycle of 12:12 h.

Cercarial emergence of *T*. *szidati* was circadian with high levels in the light period in both types of experiments and laboratory conditions, although being variable for individual replicates (Table 1; Fig 2A–2C of “peak emission experiments” in different conditions). The mean daily emergence rate was 1,117 cercariae snail^-1^ day^-1^ in the four experiments performed in July with a maximum of 4,560 cercariae per day. Cercarial productivity in the single experiment carried out in September was much higher in all snails and the mean emission rate was 10,140 cercariae snail^-1^ day^-1^, reaching a maximum of 29,560 cercariae per day. The mean daily emergence pooled across all five experiments in both months was 2,621 cercariae snail^-1^ day^-1^. Total and mean numbers of cercariae emitted for each type and day of a given experiment are shown in Table 2. Four snails died during the course of the experiments in July or before the experiments in September, resulting in a reduced number of replicates in the latter setup. Comparison of cercarial outputs recorded of snails with single and double infections in September did not detect any significant difference, although the emission from doubly infected snails appeared to be lower (t = 2.13; p = 0.07).

**Table 2:** Numbers of cercariae of *Trichobilharzia szidati* released from naturally infected *Lymnaea stagnalis* per day.

Month of experiment	Type of experiment	Day of experiment	Range	Mean±SD	Total
**July**	Daily output experiment in laboratory (DJ-L; n = 13)	Day 1	330–4,560	1,193±1,083	15,510
		Day 2	300–1,440	808±397	10,500
		Day 3	360–3,000	1,338±789	17,400
	**Total**				**43,410**
	Peak output experiment in laboratory (PJ-L; n = 11)	Day 1	600–2,520	1,181±614	12,990
		Day 2	820–2,510	1,436±473	15,800
		Day 3	810–2,600	1,619±587	17,810
	**Total**				**46,600**
	Peak output experiment in climate chamber (PJ-C; n = 11)	Day 1	530–3,230	1,849±881	18,490
		Day 2	350–1,650	777±487	7,770
		Day 3	240–1,730	881±543	8,810
	**Total**				**35,070**
	Daily output experiment in climate chamber (DJ-C; n = 10)	Day 1	140–2,700	1,238±892	12,380
		Day 2	120–2,320	894±761	8,940
		Day 3	40–970	436±285	4,360
	**Total**				**25,680**
	**Pooled data (mean)**			**1,117**	
**September**	Peak output experiment in climate chamber (PS-C; n = 9)	Day 1	4,720–29,560	13,588±7,618	122,290
		Day 2	3,710–24,070	11,494±7,669	103,450
		Day 3	2,520–10,050	5,338±2,964	48,040
	**Total**				**273,780**
	**Pooled data (mean)**			**10,140**	
**July & September**	**Pooled data (mean)**			**2,621**	

Range and total numbers of cercariae are given for each day of experiment and pooled across snail individuals (number of snails used in a given experiment is indicated by “n” in parentheses). Mean (± standard deviation, SD) represents numbers of emerged cercariae snail^-1^day^-1^. In September, only one experiment was performed.

Based on our live photographs we calculated the total volume of an individual cercaria of *T*. *szidati* to 0.0039 mm^3^ (summing volume of cercarial body: 0.0022 mm^3^, tail stem: 0.0015 mm^3^, and furcae: 0.0002 mm^3^) and estimated its mass to 0.0043 mg. Applying data on average output rates from our emission experiments pooled across all experiments conducted in both months (2,621 cercariae snail^-1^day^-1^) resulted in an estimate of 4.8 g of cercariae of *T*. *szidati*, which are emitted into an ecosystem during the life span of an individual snail, a value equivalent or even exceeding the weight of snail’s own body (range of soft tissue snail mass of 2.0–4.5 g, mean across 30 uninfected snails of 2.9 g). Based on snail density data and a *T*. *szidati* prevalence of 5%, the total annual parasite biomass in the large fishpond (47.09 ha) sums up to 561 kg. Since prevalence of *Trichobilharzia* spp. in these highly eutrophic ponds occasionally reaches more than 40% [15,37], we calculated a possible cercarial biomass in the pond of up to 4.65 tons per year.

### Chronobiology

**Fig 3:**
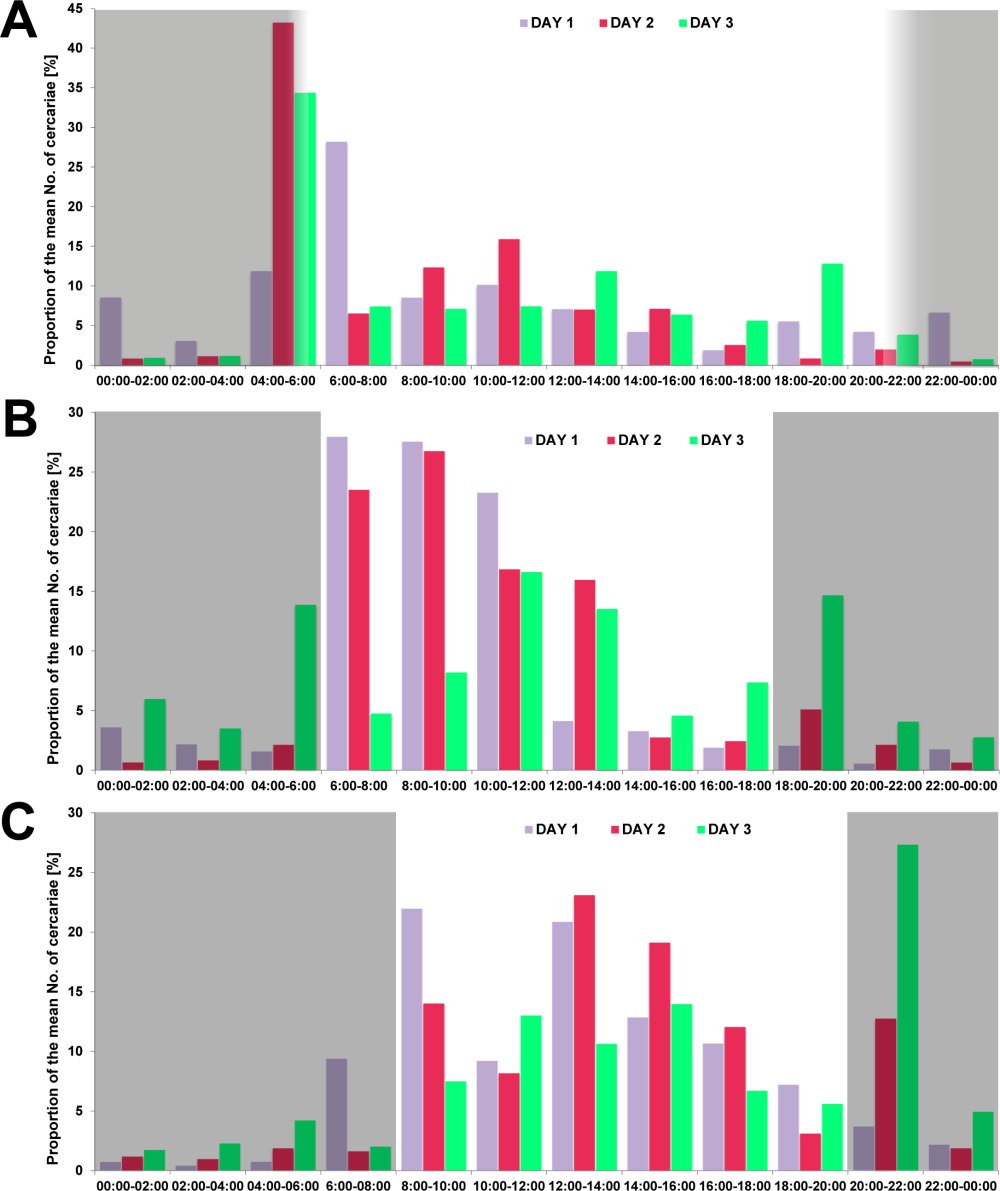
Proportions of emerged cercariae of *Trichobilharzia szidati* from *Lymnaea stagnalis* during two-hour interval. Plots showing the emerged cercariae for each day as averaged proportions of the total numbers emerged within 24 hours across all replicate snails. Grey areas indicate dark periods of the day. **(A)** The “peak emission experiment” (PJ-L) in July under laboratory conditions at room temperature (19.6°C-20.4°C) and natural photoperiod regime (light dark cycle of 16:8 h) with highest cercarial emission corresponding to sunrise at 5:30. **(B)** The “peak emission experiment” (PJ-C) in July under standard thermal conditions in a climate chamber (20°C) and light dark cycle of 12:12 h showing high cercarial emission after onset of illumination at 6:00. **(C)** The “peak emission experiment” (PS-C) in September under standard thermal conditions in a climate chamber (20°C) and light dark cycle of 12:12 h showing high emission rates after the onset of illumination at 8:00 but high variability in the emergence patterns during a day.

Although there were variations in the cercarial emission between replicates and days of experiments, clear peaks were observed in the morning hours after the onset of illumination (Table 3, Figs 2 and 3). These morning peaks were confirmed by the “daily output experiments” with cercarial counts carried out every four hours. Again, the highest numbers of emitted cercariae occurred during an interval from 8:00 to 12:00. In addition to determining emission peaks, the “peak output experiments” revealed a complex intraspecific variation in cercarial emergence depending on experimental conditions. Under natural photoperiodic regime (light dark cycle of 16:8 h in the experiment PJ-L) the emission increased rapidly with sunrise around 5:30 (Fig 3A). While the highest proportion of cercariae was released in a single two-hour interval from 4:00 to 6:00 across all replicates and three experimental days, i.e. on average 30% (range of 34–43% with a maximum of 74%) of the total number emerged within 24 hours, cercariae emitted in the remaining time units did not exceed 14% during a day. Under controlled conditions in a climate chamber (light dark cycle of 12:12 h in the experiment PJ-C) the initial peak appeared after the light was switched on at 6:00 (Fig 3B). The highest proportion of emitted cercariae under these controlled laboratory conditions was observed for two experimental days between 6:00 and 10:00 with an average range of 24–28% of cercariae released from all snails (maximum range of 42–57%). In most of the remaining two-hour intervals mean numbers of emerged cercariae remained below 4%. The cercarial output in the experiment performed in September (PS-C) showed greater variability in relation to both time units and days of the experiment compared to the July experiment (PJ-C) under similar laboratory conditions (Fig 3C). Altogether, on average 18% of cercariae (pooled across replicates and days) were released between 12:00 and 14:00 (11–21% range with maximum of 38%). Moreover, means of 22% (38% maximum) from 8:00 to10:00 and 27% (65% maximum) from 20:00–22:00 were emitted on day one and day three, respectively.

**Table 3:** Numbers of cercariae of *Trichobilharzia szidati* released from *Lymnaea stagnalis* per two-hour interval.

Month	Type of experiment	Day of experiment	Time/Number (mean) of emerged cercariae per two-hour interval											
			00:00–02:00	02:00–04:00	04:00–06:00	06:00–08:00	08:00–10:00	10:00–12:00	12:00–14:00	14:00–16:00	16:00–18:00	18:00–20:00	20:00–22:00	04:00–06:00
**July**	**Peak output experiment in laboratory (PJ-L; n = 11)**	Day 1	950 (86)	390 (35)	1,430 (130)	3,750 (341)	1,230 (112)	1,670 (152)	870 (79)	500 (45)	210 (19)	780 (71)	450 (41)	1,430 (130)
		Day 2	120 (11)	180 (16)	7,070 (643)	1,040 (95)	1,860 (169)	2,400 (218)	1,140 (104)	1,050 (95)	420 (38)	150 (14)	320 (29)	7,070 (643)
		Day 3	160 (15)	220 (20)	5,840 (530)	1,330 (121)	1,340 (122)	1,320 (120)	1,880 (171)	1,140 (104)	1,030 (94)	2,690 (245)	720 (65)	5,840 (530)
		**Total**	**1,230 (112)**	**790 (72)**	**14,340 (1,304)**	**6,120 (556)**	**4,430 (403)**	**5,390 (490)**	**3,890 (354)**	**2,690 (245)**	**1,660 (151)**	**3,620 (329)**	**1,490 (136)**	**14,340 (1,304)**
	**Peak output experiment in climate chamber (PJ-C; n = 11)**	Day 1	500 (50)	400 (40)	220 (22)	5,120 (512)	5,830 (583)	4,200 (420)	760 (76)	540 (54)	240 (24)	290 (29)	90 (9)	220 (22)
		Day 2	50 (5)	50 (5)	160 (16)	1,400 (140)	2,220 (222)	1,640 (164)	1,130 (113)	310 (31)	250 (25)	370 (37)	140 (14)	160 (16)
		Day 3	460 (46)	290 (29)	1,080 (180)	350 (35)	660 (66)	1,650 (165)	1,570 (157)	430 (43)	460 (46)	1,390 (139)	270 (27)	1,080 (180)
		**Total**	**1,010 (92)**	**740 (67)**	**1,460 (133)**	**6,870 (625)**	**8,710 (792)**	**7,490 (681)**	**3,460 (315)**	**1,280 (116)**	**950 (86)**	**2,050 (186)**	**500 (70)**	**1,460 (133)**
**September**	**Peak output experiment in climate chamber (PS-C; n = 9)**	Day 1	810 (90)	530 (59)	880 (98)	12,180 (1,353)	28,060 (3,118)	14,020 (1,558)	25,470 (2,830)	15,260 (1,696)	11,110 (1,234)	7,510 (834)	4,230 (470)	880 (98)
		Day 2	1,080 (120)	950 (106)	1,140 (127)	1,100 (122)	16,600 (1,844)	9,400 (1,044)	22,810 (2,534)	17,650 (1,961)	12,810 (1,423)	3,160 (351)	14,800 (1,644)	1,140 (127)
		Day 3	880 (98)	830 (92)	1,780 (198)	570 (63)	3,430 (381)	5,420 (602)	5,300 (589)	6,670 (741)	3,380 (376)	3,060 (340)	14,090 (1,566)	1,780 (198)
		**Total**	**2,770 (308)**	**2,310 (257)**	**3,800 (422)**	**13,850 (1,593)**	**48,090 (5,343)**	**28,540 (3,204)**	**53,580 (5,953)**	**39,580 (4,398)**	**27,300 (3,033)**	**13,730 (1,526)**	**33,120 (3,680)**	**3,800 (422)**

Total and mean (in parenthesis) numbers of cercariae released by a given number of snails used in each experiment (indicated by “n” in parentheses) per day for each two-hour interval. In September only one experiment was performed.

**Fig 4:**
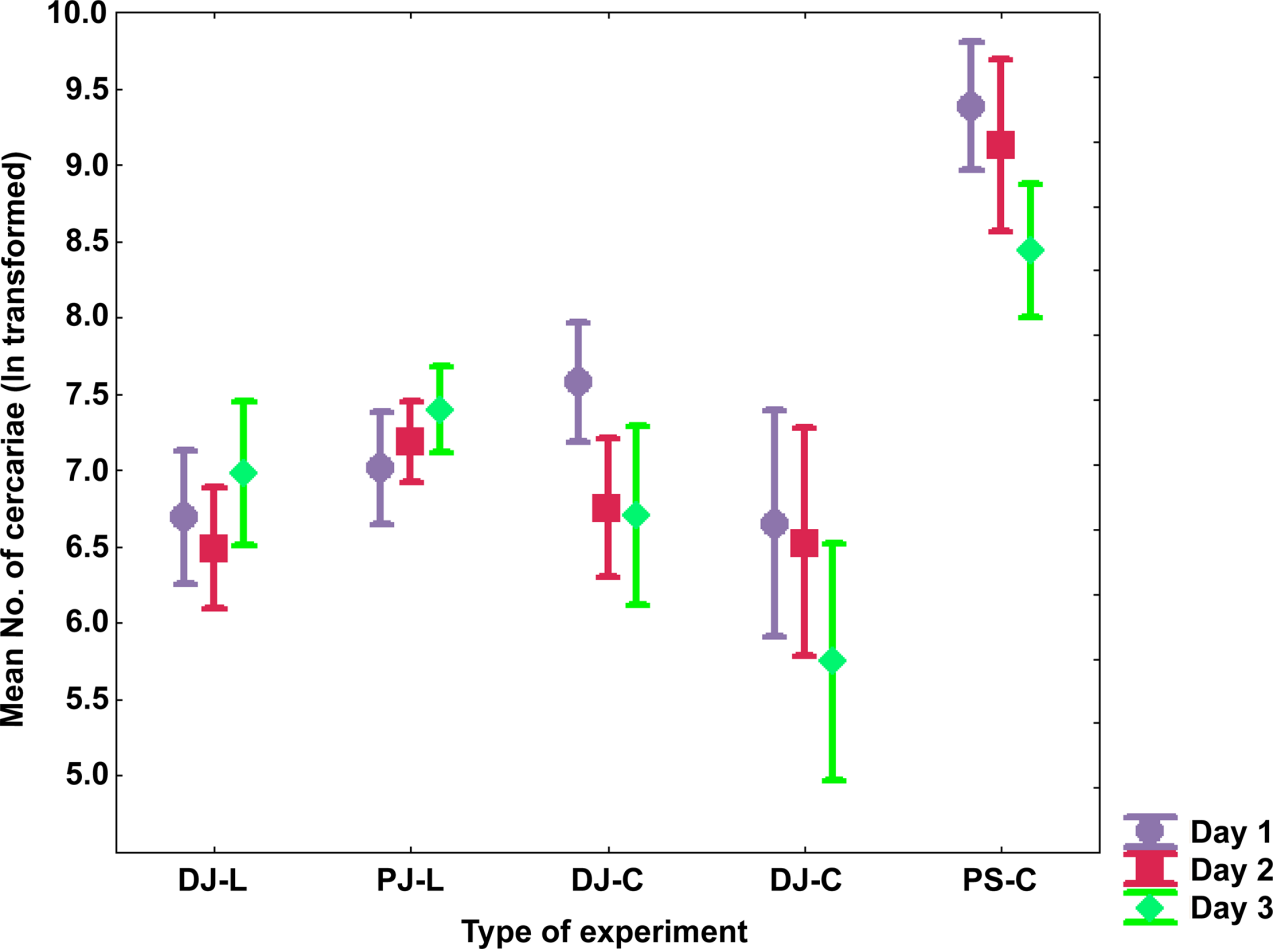
Mean number of cercariae of *Trichobilharzia szidati* (ln-transformed) from *Lymnaea stagnalis* in five emission experiments. Statistical comparison using Repeated Measures Anova showing significant differences in cercarial emission rates between four experiments performed in July (DJ-L, PJ-L, PJ-C, DJ-C) and one experiment in September (PS-C), with significantly higher cercarial emission rates in September reaching up to 29,560 cercariae per snail and day (mean 10,140 snail^-1^day^-1^). Cercarial emission followed similar pattern among days of experiments. Vertical bars denote 0.95 confidence intervals.

Following Spearman’s rank correlation analysis carried out separately for each experiment, no significant correlation between cercarial output and snail shell height (ln-transformed; mean of 42.2 mm for snails entering experiments in July and 47.5 mm for snails in September) was detected (all p>0.05). A statistical assessment of cercarial emission rates between experiments revealed significant differences between the four experiments and in relation to day of experiment, which were performed in July and one experiment in September with distinctively higher cercarial emission rates in September reaching 29,560 cercariae per snail per a day (REP-ANOVA F_(8, 64)_ = 7.86, p<10^−4^) (Tables 1 and 2, Fig 4). There was a striking overall seven-fold increase in cercarial emission of *T*. *szidati* in September, ranging from 2 to 22-fold increase across snail individuals (Table 4). Moreover, within experiments carried out in July, cercarial numbers significantly differed between DJ-C and the two “peak output experiments” (p = 0.001 for PJ-L *vs* DJ-C; and p = 0.02 for PJ-C *vs* DJ-C), probably due to the low numbers of replicate snails (Tables 1 and 2, Fig 4). Furthermore, we found significant differences in cercarial emergence in different time intervals and between days of experiment when comparing cercarial output between all three “peak output experiments” (PJ-L, PJ-C and PS-C). While different light and laboratory conditions of the two “peak output experiments” in July (PJ-L and PJ-C) did not affect the overall cercarial emission rates (Fig 2), significant differences were detected in relation to two-hours intervals between PJ-L and PJ-C (REP-ANOVA F_112, 209)_ = 4.16, p<10^−4^) and also between all three “peak output experiments” (REP-ANOVA F_(22, 297)_ = 11.31, p<10^−4^) (Table 3). However, no effect of day of experiment was detected between PJ-L and PJ-C in July (REP-ANOVA F_(2, 38)_ = 1.08, p = 0.35) indicating that cercarial emergence followed similar patterns between days in both experiments. When comparing all three “peak output experiments”, emission significantly differed among days due to the distinctly higher rates and longer release throughout the day in September (PS-C) (REP-ANOVA F_(4, 54)_ = 5.01, p = 0.002).

**Table 4:** Multiple increase in emission of cercariae of *Trichobilharzia szidati* from naturally infected *Lymnaea stagnalis*.

Snail code	Peak output experiment in laboratory (PJ-L)	Peak output experiment in climate chamber (PJ-C)	Peak output experiment in climate chamber (PS-C)	Multiple increase (PJ-L *vs* PS-C)	Multiple increase (PJ-C *vs* PS-C)
	Total number	Total number	Total number	Total number	Total number
L1	5,100	6,570	45,640	8.9	6.9
L2	6,480	7,180	39,010	6.0	5.4
L3	3,570	2,000	14,840	4.2	7.4
L4	3,900	1,770	38,350	9.8	21.7
L5	2,710	3,440	31,410	11.6	9.1
L6	4,940	6,250	53,840	10.9	8.6
L7	5,660	2,880	11,120	2.0	3.9
L9	3,830	4,370	23,980	6.3	5.5
L11	3,020	3,170	15,590	5.2	4.9
**Total**	**39,210**	**37,630**	**273,780**	**7.0**	**7.3**

Comparison of total numbers of emerged cercariae per snail pooled across three days of each experiment (i.e. 72 hours) between two peak output experiments performed in July and one in September. Only nine snails survived to September.

## Discussion

The present experimental study is the first providing comprehensive insights into the total production and patterns in cercarial emergence of the most common causative agent of swimmer’s itch in Europe, bird schistosomes of the genus *Trichobilharzia*, and allows a better understanding of the parasites’ ecological relevance and epidemiological consequences. Although it is often noted that *Trichobilharzia* cercariae are produced in high quantities [18], only few studies concern the total or average numbers of cercariae released from individual snail hosts. However, these data are rather ambiguous and often not easily comparable. In the first description of *T*. *szidati*, Neuhaus [22] estimated the number of *T*. *szidati* cercariae released from infected *L*. *stagnalis* over a period of 16 to 19 days to be 8,000 to 10,000, whereas Żbikowska [38] and Sluiters [24] described much higher productions of *T*. *ocellata* (syn. *T*. *szidati*; see [39]) cercariae from *L*. *stagnalis*, ranging from 7,500 to 10,700 per week. The most accurate descriptions from experiments on the emergence pattern of *T*. *ocellata* cercariae from *L*. *stagnalis* show a variation of 0–3,069 cercariae released during a 36-hour period and identify strong peak activities in the first hours after light exposure [23].

Our results reveal a high variability in cercarial productivity of *T*. *szidati* between individual replicates, days, two-hour intervals, type of experiment and between the different months of the experiments. The mean daily emergence rate of 1,117 cercariae snail^-1^ day^-1^ recorded in the experiments in July falls into the lower range of the weekly emission numbers described by Sluiters [24] and Żbikowska [38]. However, the cercarial productivity of individual snails was highly variable and ranged from 40 to 4,560 cercariae, which matches the observations of Anderson et al. [23]. In contrast, the extremely high emission rates in September (mean 10,140 snail^-1^ day^-1^, maximum of 29,560) have not been reported before and allow more precise estimates of the parasites’ contribution to ecosystem biomass, and better assessments of swimmer’s itch infection risks. Temperature is a major factor influencing the development of trematodes in their snail host and the production of cercariae [14,26], but room temperature was similar across all experiments (20°C in the climate chamber and 19.6–20.4°C in the air-conditioned laboratory) and does not play a role in our results. The main difference between the experiments in the climate chamber in July and September was the shift in the light regime from 6:00–18:00 (July) to 8:00–20:00 (September). However, this does not account for the higher emission in the latter experiment, since Anderson et al. [23] found no differences in emergence patterns, even if the lightning regime was reversed. Since experimental setup and all other conditions in the climate chamber (handling, length of illumination, water and air temperature) were the same in the peak output experiments, the seven-fold increase in cercarial emission of *T*. *szidati* in September can probably be best explained by intra-molluscan development cycles of sporocyst microhemipopulations in which sporocysts producing cercariae alternate with sporocysts producing daughter sporocysts, which have been described to take 35–40 days in schistosomes [40,41]. Sluiters et al. [24] describe two peaks in cercarial production of *T*. *ocellata* ca. 40 days apart. In our case there were 64 days between experiments in July and September and it is possible that we hit a production peak during that time after the newly formed sporocysts with cercariae matured. Since all snails were sampled at the same small water body at the same time and are of similar size/age, it is likely that they were infected at about the same time, explaining the similar pattern across all snails. Periods with high and low cercarial emission rates with progressive increase in emission to a maximum at the end of the four-week study have also been attributed to the variations occurring at the level of intramolluscan populations for other single snail-trematode system [42]. Taskinen [29] also recorded a gradual increase in the mean daily cercarial emission of the bucephalid trematode in a freshwater unionid clam for several months from July to October with even 74-fold emergence increase; however, although experiments were performed in the laboratory, clams were kept under natural conditions and such increase in cercarial production was attributed to increase in water temperature of the river. Cercarial emergence is known to be controlled endogenously, especially for freshwater species, when it coincides with activity rhythms of next hosts in their life cycle [12]. A similar pattern may possibly occur not only for the timing of emergence at a particular time of day but also for the amount of released cercariae of *T*. *szidati* to maximize transmission success when the chances of encountering the target hosts are highest. Hence, the period of the highest productivity of cercariae of *T*. *szidati* during the year could potentially be directly linked to mating and breeding behavior of ducks, the definitive hosts. The innate internal clock for progressive cercarial emergence might be set up for a period when sufficient populations of hosts-ducks are available. Furthermore, schistosome infection retaining in overwintering snails and renewal of cercarial release once the ambient water temperature exceeds a certain threshold [21] in spring increases the probability of contact with hosts to provide the parasite good opportunities for transmission and thus ensure continuation of their life cycle within one ecosystem in a relatively short time. This can be a result of long co-evolution and adaptation of parasites to hosts even if hosts are not present, similar to the emergence peaks which we observed in morning hours.

Another explanation for the distinctive high emission rates observed in September might be the size of snails. It is known that larger snails produce larger amounts of cercariae [11,13]. The difference in the mean shell size of replicates between the beginning of our experiments in July and September was ca 5 mm (42.2 *vs* 47.5 mm). The resources available for parasites from the hosts became greater than at the start of our study and this may also account for the increase in produced cercariae in the following experiment in September. It is likely that all mechanisms mentioned above contribute together to the effectiveness of host-parasite encounters.

Five snails in the September experiment showed patent double infections with other trematode species (four snails with *Diplostomum pseudospathaceum* and one snail with *Plagiorchis elegans*), both are being frequently found in *L*. *stagnalis* [43]. Although some of these snails showed lower *T*. *szidati* emissions (see Table 1), there was no significant difference in overall productivity or emergence patterns. Double infections, and the subsequent elimination of less dominant or subordinate trematode species as a result of interspecific competition, may be common in natural waters, especially in small eutrophic ponds with high trematode prevalence and colonization rates [35,44]. The occurrence of double infections should therefore make the results more comparable to natural conditions and no snails with double infections were excluded. Variations in cercarial production among snails can also be due to a number of reasons, ranging from slight differences in the intra-molluscan development cycles described above to individual snail metabolism [45,46] or the initial miracidial dose [24].

Parasites are usually much smaller than their hosts. While this is certainly true at the level of the individuum, the combined biomass of parasites contributes significantly to the total biomass in an ecosystem [3]. Only few studies quantified trematode biomass in an attempt to assess the potential function and contribution of free-living stages of parasites to the energy flow in ecosystems. Kuris et al. [3] estimated that the annual production of trematode cercariae in the Carpinteria Salt Marsh represented a biomass greater than that of other parasites and even of birds, the top predators in this system. They concluded that the presence and abundance of a certain type of organism in a given territory may be dependent on the activity of trematodes, thus stressing their importance in structuring aquatic food webs and energy transfer. Thieltges et al. [11] used published data on the cercarial output rates and calculated the annual production of cercariae comparable to biomass estimated for free-living benthic marine invertebrates. Most recently, in freshwater pond ecosystems in California, the trematode productivity and cumulative biomass which was equal or exceeded the biomass of the most abundant insect groups was comparable to those estimated for marine and estuarine systems [7].

Based on the information from our cercarial emission experiments, we can make sound estimations of the biomass productivity of *T*. *szidati* cercariae at two levels, i) the cercarial productivity of an individual snail during its lifetime, and ii) the annual contribution of *T*. *szidati* cercariae to an ecosystem’s biomass. The cercarial production of 4.8g of a single snail during its lifetime underlines the ecological importance of these trematodes at the host-parasite level. By relocating the snail’s reproductive resources, and thereby castrating the host [17,18], the parasites are able to transfer a substantial amount of biomass into cercarial production. Besides being “hands inside of a puppet” that take up a large amount of the host’s soft tissue mass (on average around 20%, [47], trematodes are able to produce a substantial amount of biomass outside the host that equals or even exceeds the weight of the soft tissue mass of the snail host during its life span.

On an ecosystem level, our results show that a single trematode species contributes a considerable amount of cercarial mass to an aquatic ecosystem during the parasite’s active period in the summer months of a year. Using a conservative estimation of a *T*. *szidai* prevalence of 5%, we calculated an annual biomass contribution of 561kg into the small fishpond. However, since prevalence of *Trichobilharzia* sp. in this pond of more than 40% have been reported [37], we end up with a possible annual cercarial production of 4.6 tons. This would equal the weight of a large Asian elephant, an illustrative figure/comparison already used to describe parasite contributions to the biomass in estuarine systems [48]. While both the elephant and the reproduction of the snail’s individual weight are certainly impressive, we have taken care to follow a rather conservative approach in our calculations. *Lymnaea stagnalis* can live up to three years [49] and may thus produce even more cercariae than estimated for the two-year lifetime we assumed. Furthermore, the body volume we calculated for *T*. *szidati* based on our measurements of live cercariae is considerably smaller than the one calculated for *T*. *szidati* by Koehler et al. [31] based on literature data (0.0039 mm^3^ *vs* 0.0068 mm^3^, respectively) resulting in possibly higher actual contribution of *T*. *szidati* to an ecosystem’s biomass than our calculations. Moreover, the mean number of cercariae (2,621 snail^-1^day^-1^) across all our experiments is low compared to the possible peaks of 29,560 cercariae per snail and day detected in September. If such peaks occur frequently, the total number of cercariae and their biomass contribution may turn out higher still. In Central European freshwater ecosystems bird schistosomes, such as *T*. *szidati*, only constitute a fraction of the diverse trematode communities which comprise a multitude of species [35,50,51], all of which contribute to the ecosystem’s biomass. Depending on the transmission strategies of the parasites, daily cercarial emission can be significantly higher than in bird schistosomes, e.g. in *Diplostomum* spp., an important pathogens of fish, with productions of up to 60,000 cercariae snail^-1^day^-1^ [52, 53], and well beyond up to 500,000 cercariae per snail and day for some species [54]. Given that *L*. *stagnalis* is known to harbour 23 species of cercariae ([55], but see [56] for the complex of species of the genus *Plagiorchis*), it is safe to assume that the overall cercarial biomass emitted into these systems is comparable to the impressive numbers recently calculated for marine [11], estuary [3] and North American freshwater ecosystems [7]. Since the majority of produced cercariae are not able to successfully infect a suitable target host as they end up as food for predators [57,58,59], contribute to the ecosystem’s detritus, or interfere passively with other organisms, they contribute significantly to the energy flow in aquatic systems [11,59,60]. Although not tested experimentally, our estimates demonstrate the significance of a single life stage of a single trematode species in the food web dynamics. The large numbers of *T*. *szidati* cercariae released into the environment show the different roles of the free-living larvae of this species that are produced in such large quantities in order to facilitate transmission to a suitable final host (Fig 1A) but may provide a strong potential infection risk for humans (Fig 1B), whilst at the same time contribute substantially to the energy flow in the aquatic system (Fig 1C and 1D). Overall, our results are transferable to any other freshwater systems in terms of expecting similar numbers of cercariae of *T*. *szidati* emitted from *L*. *stagnalis* to the environment.

It is worth mentioning that there are alternative paths operating at the trematode infracommunity level within individual snail in food webs, which may also affect the magnitude of parasite biomass. In addition to the cercariae released into the aquatic environment, the parasite stages within an individual snail host, i.e. the rediae and sporocysts that produce cercariae, further contribute to the overall parasite biomass and can play an important role in the energy flow in ecosystems, e.g. in concomitant predation when the parasites are indirectly eaten together with their hosts [57,59], interspecific competition within snails, eventually leading to an exclusion of subordinate species [44,61], or parasite-induced or natural death of snails.

Emergence of *Trichobilharzia* cercariae has been shown to occur in the first hours of light exposure [23] and was determined to peak early in the morning hours between 9 and 11am [24]. Our results clearly confirm the strong peak activities in cercarial emission after the onset of light exposure described by Anderson et al. [23], both in controlled environments and under natural conditions with gradually increasing daylight. In all experiments, high emission rates were observed immediately at the beginning of the light phase, following a low-emission dark regime. These patterns suggest a synchronization of cercarial emission with the daily activity patterns of the definitive hosts, ducks that also show strong diurnal patterns with the highest activities around sunrise and sunset [62]. Such a synchronization of cercarial release with host-time can be explained as an adaptive behavior enhancing the probability of transmission success of the short-lived cercariae to their hosts [12]. This is also a common feature with mammalian schistosomes in which cercarial emergence varies on a circadian cycle and is associated with definitive host availability [63,64,65]. The morning emission of cercariae was most prominent in the peak output experiment conducted in the laboratory with natural lightning conditions (Figs 2A and 3A). The experiments conducted in the climate chamber showed longer windows of continuous emission during the light period (Figs 2B and 2C and 3B and 3C). This may be due to the exposure to a direct light source (halogen lamp) in the climate chambers in contrast to the changing intensity of illumination in the laboratory throughout the day. Since the windows in the laboratory were facing north, snails were mostly exposed to indirect sunlight, whereas the climate chamber experiments simulate conditions similar to an unshaded area during a summer day, possibly explaining the longer emission patterns. Furthermore, we observed small emission peaks in the climate chamber experiments during the dark phases (see Figs 2B and 2C and 3B and 3C). It is possible that snails were accidentally exposed to indirect light when the door to the climate chamber was briefly opened during the handing of the snails. This would suggest that only very brief light impulses are sufficient to trigger cercarial emission and show a high flexibility of the parasites to react to environmental changes, e.g. short sunny phases in cloudy weather.

Besides the biomass contribution of *T*. *szidati* and ecological importance of these parasites in food webs, our results may help to give better estimations of the risks of swimmer’s itch that can be caused by bird schistosomes. The exceptionally high emission rates of cercariae in the September experiment highlight why infections in humans are typical, even in regions where prevalence of bird schistosomes is very low. Furthermore, the more cercariae emitted into the environment, the higher the chances that they are spread to different areas of the water body as they can be transported by winds and currents for several kilometres [66,67]. A single infected *L*. *stagnalis* snail appears to be enough to create a potential ‘infection hot spot’ of swimmer’s itch, e.g. if about 29,500 cercariae are released at a shallow area frequented by many swimmers during a day. *Trichobilharzia szidati* can be considered the most prevalent etiological agent of swimmer’s itch, as *L*. *stagnalis* is the most common, abundant and widely distributed pulmonate snail in the Holoarctic and typically occurs in stagnant waters [32,68] such as ponds, which are commonly used for bathing activities in Europe and whose eutrophic status favours parasite transmission and disease outbreaks [15].

## Conclusions

The results of our study show the large cumulative biomass of *T*. *szidati* cercariae both on the individual host and the ecosystem level and thus highlight the parasites’ significant contribution to the energy flow in aquatic systems. We can confirm strong peak activities in cercarial emission as a result of illumination but were able to show that emission patterns can be flexible and large quantities of cercariae can be released throughout the course of a day, if the snails are exposed to light. Therefore, while the early worm catches the bird, it tries to do so many times. Although bird schistosomes constitute only a fraction of the diverse trematode communities in the studied aquatic ecosystems, their infective stages, the cercariae, but can still pose a considerable risk of swimmer's itch due to the high number of cercariae emitted from infected snails.
